# Comprehensive profiling of *Rhodiola rosea* roots and corresponding products: phytochemical insights and modulation of neuroinflammation in BV2 microglial cell model

**DOI:** 10.3389/fphar.2025.1608767

**Published:** 2025-07-02

**Authors:** Małgorzata Kołtun-Jasion, Karolina Czerwiec, Andrzej Parzonko, Aleksandra Bakiera, Marcin Ożarowski, Anna Karolina Kiss

**Affiliations:** ^1^ Department of Pharmaceutical Biology, Faculty of Pharmacy, Medical University of Warsaw, Warsaw, Poland; ^2^ Institute of Natural Fibres and Medicinal Plants, National Research Institute, Poznań, Poland

**Keywords:** *Rhodiola* L., golden root, rosavin, adaptogens, dietary supplement, microglia, neuroinflammation

## Abstract

**Introduction:**

*Rhodiola rosea* L., mainly known within the medicinal plant industry as golden root, Arctic root, or rose root, derives its name from its economic significance, distinctive morphology, and restricted geographical distribution. Extracts from *Rhodiola rosea* roots/rhizomes are widely used across Europe and Asia as ingredients of traditional herbal medicines and dietary supplements, with numerous claims regarding their adaptogenic effects. With the growing demand for pharmaceutical products that relieve stress-related fatigue and exhaustion, driven by technological advancements and increasing psychophysical challenges, *R. rosea* has become a highly sought-after resource. However, this heightened demand has also increased the risk of adulteration and the proliferation of low-quality products on the market. The reproducible efficacy and quality of *R. rosea* preparations are largely dependent on the variable content of key active compounds, such as rosavin, which directly influence product quality. The rapid expansion of the dietary supplement market, coupled with insufficient quality verification of products entering the market, underscores the need for rigorous identification and quality assessment of these products.

**Methods:**

This study aimed to perform a phytochemical analysis of 13 dietary supplements claiming to contain *R. rosea* using HPTLC and LC-MS techniques and to correlate these findings with their anti-inflammatory activity in an LPS-stimulated BV2 microglial cell model, *in vitro*.

**Results:**

Our study indicates that nearly 60% of the tested preparations did not contain the declared amount of *Rhodiola rosea* roots/rhizomes or the characteristic marker compounds associated with this species. Furthermore, rosavin was detected in only 9 out of the 13 analyzed products, with 4 of these containing only trace amounts of this marker compound. Misidentification of *R. rosea *was most frequently observed among tablet and capsule formulations, whereas products in the form of cut raw material exhibited the highest quality. Moreover, rosavin significantly and dose-dependently inhibited the secretion of interleukin-6 (IL-6) and tumor necrosis factor-alpha (TNF-α) in lipopolysaccharide (LPS)-stimulated microglial cells.

**Discussion:**

The identification of *R. rosea* in only 40% of the preparations underlines that rigorous control and standardisation of herbal supplements are crucial to understanding their therapeutic activity and preventing adulteration.

## Graphical Abstract

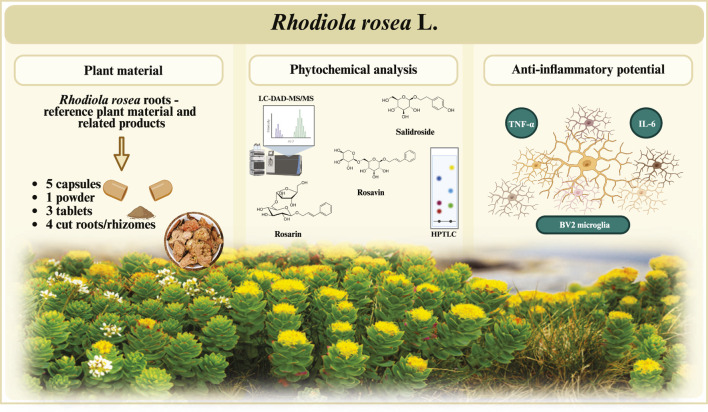

## 1 Introduction

*Rhodiola rosea* L., [Crassulaceae, syn. *Sedum rhodiola* Vill] is affiliated with a group of perennial shrubs commonly associated with subarctic and alpine areas of eastern Siberia. Currently, *Rhodiola rosea* is found in hard-to-reach rocky terrains, sea cliffs and mountains of the northern hemisphere, ranging from the low Arctic to the high temperate zones of Asia, Europe, and North America (Scandinavia, Iceland, Greenland, Alaska or Canada), making the world supply from wild populations on an industrial scale extremely limited (Blomkvist et al., 2009).

To date, 74 accepted species have been identified within the *Rhodiola* genus. Many of them are used in national systems of traditional medicine, including *Rhodiola heterodonta* (Hook.f. and Thomson) Boriss, *Rhodiola quadrifida* (Pall.) Fisch and Mey, *Rhodiola semenovii* (Regel and Herder) Boriss, *Rhodiola kirilowii* (Regel) Maxim., and *Rhodiola crenulata* (Hook.f. and Thomson) H.Ohba. Of all these species, *R. rosea* is the one most studied for its phytochemical properties as well as its use in medicine and therapy. *R. rosea* is one of the main representatives of the group of plant adaptogens used for medicinal purposes linked to stress and age-related impairments of cognitive functions as a tonic and immunomodulating therapeutic agent (European Food Safety Authority (EFSA), 2012). The most valuable parts of the plant used in therapy are *R. rosea* roots and rhizomes, of a golden metallic colour, harvested after at least a 5-year vegetative period (Galambosi, 2006).

*R. rosea* has a long history of use as a medicinal plant, appearing in reports from Asian and European countries. The therapeutic use of *Rhodiola rosea* roots traces back to Tibetan Traditional Medicine (TTM), which has influenced its more recent use in Traditional Chinese medicine (TCM), where it was employed to treat altitude sickness, fatigue, and mood disorders, as well as headaches, diarrhoea, skin conditions and swelling, typically in the form of infusions and tinctures (Tao et al., 2019).

The European Medicines Agency classifies the dry extract (DER 1.5–5:1) obtained from *R. rosea* roots and rhizomes as a traditional herbal medicinal product used for the prevention and temporary relief of fatigue and sensation of psycho-physical weakness (European Medicines Agency, 2012). Currently, in the European Union, *R. rosea* preparations are used as active ingredients in registered traditional herbal medicinal products (HMP) and as ingredients in dietary supplements, suggesting potential benefits on memory, learning, and attention. Supplements containing *Rhodiola* are recognised as beneficial for managing physical and mental stress in the European Food Safety Authority’s (EFSA) consolidated list of Article 13 health claims (European Food Safety Authority, 2012). Moreover, *R. rosea* root extracts can be found in “functional foods”, sports and energy drinks, alcoholic beverages, as well as the cosmetic industry (European Commission, 2020; Lamadrid et al., 2019).

The multidirectional properties of *R. rosea* - concerning its antibacterial, anxiolytic, antioxidant, immunomodulating, cardio and neuroprotective, as well as nootropic effects are strongly correlated with the rich phytochemical composition of its underground organs (Lee et al., 2013; Zhang et al., 2022). Chemical investigation on this plant revealed approximately 150 chemical entities, encompassing phenylethanoids (i.e., salidroside with its aglycone–tyrosol and its derivatives), phenylpropanoids (i.e., rosin, rosavin, rosarin), and flavonoids characteristic of this species (herbacetin, gossypetin and kaempferol derivatives), as well as cyanogenic glycosides, gallic acid derivatives and proanthocyanidin constituents among others (Alperth et al., 2019). Although it is still not entirely clear which compounds are responsible for the adaptogenic effects of the raw material, the observed effects have been considered to be mainly based on phenylethane and phenylpropane derivatives. The latest edition of the European Pharmacopoeia (11th edition, 2023) emphasises the quality assessment of *R. rosea*-based products by considering the content of marker compounds, salidroside and rosavins, in a ratio not lower than 1:3. In turn, the United States Pharmacopeia guidelines assess the quality mark for the raw material as containing not less than 0.3% of the phenylpropanoid glycosides rosarin, rosavin and rosin (these three compounds are also collectively referred to as ‘rosavins’) calculated as rosavin, and not less than 0.08% of salidroside, calculated on a dry weight basis (United States Pharmacopeial Convention, 2017; Ezra Bejar and John, 2017). Salidroside has been identified in several species of the *Rhodiola* genus, including *R. crenulata*, *R. rosea*, *R. sachalinensis*, and *R. kirilowii*, while rosavin has been isolated specifically from the roots of *R. rosea* and *R. sachalinensis* (in smaller amounts) (Booker et al., 2016; Nakamura et al., 2008). Despite the presence of salidroside in most species of the *Rhodiola* genus, its relatively low content in *R. rosea* does not allow for its unequivocal recognition as a marker compound for this species. Consequently, monitoring the presence of rosavin, as a distinctive chemotaxonomic marker, seems to be considered synonymous with the high quality of the tested preparations.

The rising demand for *R. rosea* has been paralleled by an increase in dietary supplements claiming its inclusion. Considering the economic advantage of producing dietary supplements over medicinal products, as well as the lack of precise guidelines for analysing their composition, proper quality control of *R. rosea* preparations poses a significant global challenge within the pharmaceutical industry. The plant’s limited native range, coupled with its difficult accessibility, prolonged growth period, and morphological similarities within the *Rhodiola* genus, has led to frequent adulteration with other species (Nikolaichuk et al., 2021). Furthermore, the adaptogenic properties attributed to *R. rosea*, together with the growing number of individuals seeking relief from excessive psycho-physical stress, fatigue, and exhaustion, have rendered *R. rosea* an endangered species in many regions. The global demand for adaptogenic medicinal plants such as *R. rosea* and its key compounds, salidroside and rosavins, has also driven an increase in preclinical and clinical studies aimed at uncovering new applications for *R. rosea* in modern phytotherapy, which has further depleted the natural resources of the raw material (Hung et al., 2011; Lu et al., 2022).

The aim of our study was to assess the phytochemical analysis of commercial products of the underground parts of *R. rosea* and/or their potential contaminants. Herein, HPTLC and LC-MS fingerprints describing the phytochemical composition of 13 preparations containing *R. rosea* unprocessed roots or their extracts were analysed, with a particular emphasis on assessing the rosavin presence within these products. The traditional use of *R. rosea* is well-documented for its adaptogenic and stimulating effects. As the search for natural substances that help the human body adapt to challenges from technological advancements and modern working conditions continues, we analysed the qualitative composition of the tested extracts, including rosavin, and correlated these findings with their anti-inflammatory effects in a BV2 microglial cell model. Incorporating the potential impact of the tested samples on inflammatory responses within a microglial model may provide a valuable tool for additional quality control of adaptogenic plant materials.

## 2 Materials and methods

### 2.1 Plant material used for the study

The plant material, including dietary supplements available in pharmacies and herbal stores, was purchased commercially. The preparations claiming to contain extracts from the roots and rhizomes of *Rhodiola rosea* were obtained as standarised/non–standarised extracts in the form of tablets (3) or capsules (5). The herbal products consisting of cut, unprocessed roots/rhizomes (4) or powdered raw material (1) were also analyzed. Reference material (*Rhodiola rosea* roots, Crassulaceae) was obtained from the Institute of Natural Fibres and Medicinal Plants crops controlled cultivation.

Detailed information on the examined preparations is provided in the Supplementary Material (Supplementary Table S1). A voucher specimen of the tested dietary supplements and plant material has been deposited in the Plant Collection of the Department of Pharmaceutical Biology at the Medical University of Warsaw (no. 80/2024). The materials used for the study are summarised in the table below (Table 1).

**TABLE 1:** Source of plant material used in the study.

Symbol	Dosage form	Formulation	Declared species	Origin
K1	Capsules	Extract standardised to 4% salidroside	*R. rosea*	—
K2		Powdered rhizomes	*R. rosea*	—
K3		Extract standardised to 3% salidroside	*R. rosea*	—
K4		Root extract 4:1	*R. rosea*	—
K5		Extract standardised to 3% rosavin and 1% salidroside	*R. rosea*	—
T1	Tablets	Powdered roots, standardised to 3,2% rosavin and 1% salidroside	*R. rosea*	—
T2		Root extract 4:1	*R. rosea*	—
T3		Extract standardised to 3% salidroside	*R. rosea*	—
H1 H2 H3 H4	Cut raw material	Cut raw material (root) Cut raw material (rhizome) Cut raw material (root) Cut raw material (root)	*R. rosea* *R. rosea* *R. rosea* *R. rosea*	Russia Poland Russia Russia
P1	Powder	Powdered roots, standardised to 3,12% rosavin	*R. rosea*	China
Reference plant material	Cut raw material	Cut raw material (root)	*R. rosea*	Poland

(—), no information regarding the origin of the raw material.

### 2.2 Preparation of extracts from investigated samples for phytochemical analysis

The contents of tablets, capsules, sachets, as well as cut roots and rhizomes, were finely ground. Extracts were prepared using 60% ethanol (Avantor Performance Materials Poland S.A., Gliwice, Poland) as the solvent, in accordance with the guidelines of the European Medicines Agency (European Medicines Agency, 2012). Considering the diverse chemical groups present in *R. rosea*, infusions were also prepared from each of the examined dietary supplements as the most widely used and most easily available form of natural raw materials administration.

Infusions were prepared by adding boiling water to the plant material at a ratio of 1:10 and left under cover for 15 min according to the method recommended by the European Pharmacopoeia. Ethanolic extracts at a concentration of 60% were prepared by adding a solvent to the finely ground and crushed material (1:5 ratio) and heating it at 95°C for 1 hour. After filtration, ethanol was evaporated under reduced pressure. The aqueous residues (from infusions and ethanolic extracts) were lyophilised to obtain dry powders.

Lyophilisates were dissolved in methanol:water (8:2, *v/v*) to reach a final concentration of 10 mg/mL. The reference substance (rosavin, Sigma–Aldrich Chemie GmbH, Steinheim, Germany) was dissolved in pure methanol to obtain a stock solution at a concentration of 1 mg/mL.

### 2.3 HPTLC apparatus and chromatographic conditions

The High-performance thin-layer chromatography (HPTLC) analysis was performed using a TLC system provided by Camag (Muttenz, Switzerland). The setup included a Linomat 5 sample applicator fitted with a 100 µL Hamilton syringe, an Automatic Developing Chamber 2 (ADC2), a Scanner 4, an Automatic Derivatizer, a Visualizer 2, and a Plate Heater III. Device operation and image analysis were managed using the WinCATS 1.4.2 software (Camag).

Silica gel 60 F_254_-coated HPTLC plates, glass-backed, measuring 20 × 10 cm (Merck, Darmstadt, Germany), served as the stationary phase. Before use, the plates were prewashed with methanol, dried for 10 min at ambient temperature, and subsequently activated at 80°C for 30 min. Extract samples (10 mg/mL) and standard solution samples (1 mg/mL) were applied in 10 µL volumes as 6 mm bands, with 12 mm spacing between tracks and distances of 12 mm and 10 mm from the side and lower plate edges, respectively.

A consistent application rate of 150 nL/s was maintained. The plates were developed using a mobile phase comprising ethyl acetate, methanol, water, and formic acid in the ratio of 77:13:10:2 (*v/v/v/v*). Plate development took place in an automatic developing chamber, which was presaturated with 10 mL of the mobile phase at a controlled room temperature of 23°C ± 2°C and a relative humidity of 40% ± 5%. The plate development process involved the following parameters: humidity control for 5 min, tank saturation for 5 min, plate preconditioning for 5 min, a mobile phase migration distance of 80 mm, and a plate drying time of 10 min. Spot visualisation was performed under white light as well as UV light at 254 nm and 366 nm. For derivatisation, plates were treated with a 1% solution of vanillin in sulfuric acid solution (Loba Feinchemie, Apolda, Germany). The derivatisation process was carried out using a CAMAG Automatic Derivatizer (set to level 3). Post–spraying, the plates were imaged again under white light and UV light at 254 nm and 366 nm.

### 2.4 UHPLC–DAD–ESI–MS/MS analysis

The Ultra-High Performance Liquid Chromatography – Diode Array Detection – Electrospray Ionization – Tandem Mass Spectrometry (UHPLC–DAD–ESI–MS/MS) analysis was performed on a UHPLC–3000 RS system (Dionex, Germering, Germany) with DAD detection and an AmaZon SL ion trap mass spectrometer with an ESI interface (Bruker Daltonik GmbH, Bremen, Germany). Separation was performed on a Zorbax SB–C18 column (150 × 2.1 mm, 1.9 μm) (Agilent, Santa Clara, California, USA). The mobile phase consisted of water +0.1% formic acid (A) and acetonitrile +0.1% formic acid (B). A gradient was applied as follows: 0–60 min, 5%–40% B. All solvents used for analysis (methanol, formic acid acetonitrile; Avantor Performance Materials Poland S.A., Gliwice, Poland) were of gradient grade. The LC eluate was introduced into the ESI interface without splitting, and the analysis was performed in negative ion modes with the following parameters: nebuliser pressure at 40 psi; drying gas flow rate at 9 L/min; nitrogen gas temperature at 300°C; and a capillary voltage of 4.5 kV. The mass scan range was set from 100 to 2,200 m*/z*. UV–VIS detection covered the 190–600 nm range. All samples were analysed using the UHPLC–DAD–ESI–MS/MS method, and the predominant substances in each extract were identified by comparing retention times and spectra (UV, MS, MS/MS) with standard and literature data.

### 2.5 Preparation of tested extracts for bioassay

Lyophilisates were dissolved in DMSO (Sigma–Aldrich Chemie GmbH, Steinheim, Germany) and then diluted with (Mg^2+^, Ca^2+^)–free PBS buffer at pH 7.4, reaching a final 1 mg/mL concentration. Control with dexamethasone (Merck, Darmstadt, Germany) as well as the reference marker–rosavin, were initially dissolved in DMSO to obtain 10 mM stock solutions, which were then diluted with (Mg^2+^, Ca^2+^)–free PBS buffer. The infusions and ethanolic extracts were tested at a concentration of 50 μg/mL. Rosavin was evaluated across a concentration range of 0.1–5 μM, while the control samples containing dexamethasone were assessed at a concentration of 20 µM. The concentration of DMSO (<0.1% in the final added quantity) did not affect the outcomes of the assays. Appropriate dilutions and final concentrations of LPS were prepared using DPBS solution.

### 2.6 Determination of BV2 cell viability

The immortalised murine microglial cell line BV2 (passages 1–4) was purchased from DSMZ–German Collection of Microorganisms and Cell Cultures GmbH. BV–2 cells (passages 1–5) were cultured in RPMI medium 1,640 (Thermo Fisher Scientific, Waltham, MA, USA) supplemented with 10% FBS and antibiotics (1% penicillin–streptomycin and 0.5% amphotericin B; PAA, Laboratories GmbH, Pasching, Austria) at 37°C, 5% CO_2_. For all experiments, BV–2 cells were incubated in RPMI 1640 medium containing 2% FBS, supplemented with antibiotics.

Cells were seeded at 4 × 10^3^ cells/ml in 96–well microtiter plates (Nunc™, Thermo Fisher Scientific) and cultivated for 24 h at standard treatment conditions. BV2 microglia were exposed to the tested extracts (or appropriate controls), with or without stimulation with LPS (100 ng/mL) from *Escherichia coli* 0111:B4 (Sigma–Aldrich Chemie GmbH, Steinheim, Germany) for 24 h. Following treatment, the cell culture medium was replaced with a culture medium containing NRU dye supplement (50 μg/mL) or MTT solution (0.5 mg/mL). Following 6 h incubation, intracellular NRU dye was extracted using a destaining solution (ethanol, water, and acetic acid, mixed in a 50:49:1 ratio). For the MTT assay, following a 4–hour incubation, the medium was removed, the cells were washed with DPBS, and the formazan crystals were dissolved in 200 μL of DMSO. Absorbance was subsequently measured at 540 nm for NRU and 580 nm for MTT assay, using a Synergy 4 BioTek microplate reader (Winooski, VT, USA).

### 2.7 TNF-α, IL-6 secretion by BV2 cells

BV2 cells were plated in 24–well plates at the density of 1 × 10^5^ cells/mL and cultivated for 24 h, at standard treatment conditions. Subsequently, the cells were treated with tested extracts (50 μg/mL) or rosavin (20 µM), followed by the stimulation with LPS (100 ng/mL). After 24 h, collected supernatants were centrifuged, and the cytokines’ release was determined by ELISA assay kits (BD Biosciences, San Jose, CA, USA) following the manufacturer’s instructions using a microplate reader. The effect on cytokine production was determined through the percentage of released cytokines relative to the LPS–stimulated control. Dexamethasone (20 µM) was used as a positive control.

### 2.8 Statistics and data analysis

The results were expressed as mean ± SEM for three independent experiments performed at least in triplicate. One–way analysis of variance (ANOVA), HSD Tukey test, and Dunnett’s test were applied to evaluate the statistical significance of the mean values, with *p < 0.05, **p < 0,01, ***p < 0.001, regarded as statistically significant. GraphPad Prism 10 was used for all analyses and graphs.

## 3 Results

### 3.1 HPTLC analysis of ethanolic extracts prepared from *Rhodiola rosea* commercial samples

**FIGURE 1:**
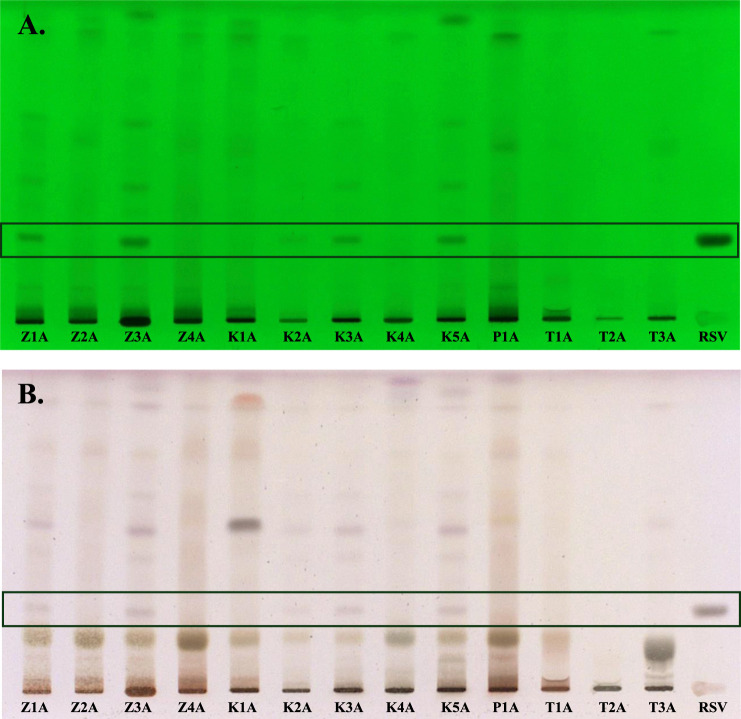
HPTLC chromatograms of the *Rhodiola rosea* dietary supplements, separated on HPTLC plates silica gel F_254_, using ethyl acetate–methanol–water–formic acid (77:13:10:2, *v/v/v/v*) mobile phase and detected at **(A)** 254 nm (before derivatization) and in **(B)** white light illumination (after derivatization). Z1A–Z4A–unprocessed raw materials of *Rhodiola rosea* roots/rhizomes; K1A–K4A–capsules; P1A–powder; T1A–T3A–tablets; RSV–rosavin.

Qualitative evaluation of the composition of the tested samples, declaring the content of *R. rosea* roots/rhizomes or their extracts, was conducted using the HPTLC method. The analysis enabled the preliminary verification of the composition of 13 dietary supplements. The major bioactive compounds of *R. rosea*, e.g., rosavin, are often used for the quality evaluation of *R. rosea* preparations (Booker et al., 2016). Notably, the analysis confirmed the presence of rosavin in 5 of the 13 tested preparations. After the development of chromatograms, the band corresponding to rosavin was observed under UV light at a wavelength of 254 nm, with an Rf value of 0.22 (Figure 1A). Further verification through chemical derivatization using vanillin in a sulfuric acid solution confirmed the presence of rosavin as a violet band (details provided in the Supplementary Figures S1, S2), which was visible in samples Z1A, Z3A, K2A, K3A, and K5A (Figure 1B). Notably, HPTLC profiling revealed that the characteristic marker compound, rosavin, was absent in 8 products claiming to contain *Rhodiola rosea*.

The HPTLC analysis also revealed that the **K1A**, **P1A**, and **T1A** samples exhibited a fingerprint inconsistent with that of *R. rosea* (e.g., **Z3A**) and were likely adulterated with other *Rhodiola* species. Additionally, two of the tested samples (**T1A** and **T2A**) showed the presence of unspecified components unrelated to *R. rosea*.

### 3.2 LC–MS analysis of *Rhodiola rosea* roots aqueous and ethanolic extract

**FIGURE 2:**
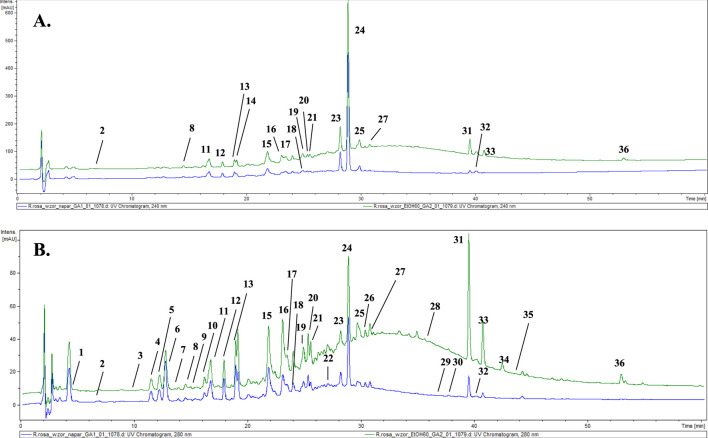
LC-DAD-MS/MS chromatograms of extracts (60% ethanolic extract–green and aqueous extract–blue) from reference plant material, recorded at **(A)** 240 nm and **(B)** 280 nm. The numbered peaks are denoted in Table 2.

From the reference material–cut, unprocessed roots of *Rhodiola rosea*, an infusion and a 60% ethanolic extract were prepared. Phytochemical analysis of the prepared solutions was conducted using the HPLC–DAD–MS/MS method. The chromatogram and the analysis of the reference extracts (60% ethanolic–green and infusion–blue), recorded at a wavelength of 240 and 280 nm, are presented below (Figures 2A,B; Table 2).

**TABLE 2:** Retention time, UV, and MS/MS data of the compounds present in the tested extract.

No	Compound	UV [nm]	Rt [min]	[M-H]^-^	Product mass peaks	Group	Ref.
1	Gallic acid	271	4.3	169.08	125	Phenolic acids	Lee et al. (2016)
2	Rhodiocyanoside A	208	6.5	304.14^b^	258.04 178.90	Cyanogenic glycosides	Alperth et al. (2019)
3	Lotaustralin	203	9.9	306.11^b^	260.08 101.18	Cyanogenic glycosides	Alperth et al. (2019)
4	Unknown	205	11.6	306.16	−	−	−
5	Unknown	267	12.3	343.11	297.05 135.03	−	−
6	Salidroside	215, 280	12.8	345.22^b^	299.02	Phenylethanoids	Alperth et al. (2019)
7	Rhodioloside D	205	13.4	395.22^b^	349.11 178.92	Monoterpene glycosides	Avula et al. (2009), Han et al. (2016b)
8	Unknown	207	14.7	293.16	−	−	−
9	Epigallocatechin	275	15.1	305.16	218.92 178.82	Catechins	Olennikov et al. (2020)
10	Olivil-*O*-glucoside	207	16.2	537.16	375.09	Lignans	−
11	Prodelphinidin B-2-*O*-gallate	209	16.7	761.18	609.18 483.08 423.10 305.04 242.93	Proanthocyanidins	Alperth et al. (2019)
12	Viridoside	263	17.9	359.31^b^	313.06 178.91	Phenylethanoids	Alperth et al. (2019)
13	Herbacetin-*O*-glucorhamnoside-*O*-rhamnoside	275	19.1	755.29	609.23 447.53 299.98	Flavonol	Chen et al. (2021)
14	Benzyl alcohol arabinosyl-glucoside	209	19.3	447.37^b^	437.16	Monoterpene glycosides	Olennikov et al. (2020)
15	Prodelphinidin B-2 di-*O*-gallate	275	21.9	913.16	761.17 608.76 423.29 304.95	Proanthocyanidins	Alperth et al. (2019), Yousef et al. (2006)
16	Epigallocatechin gallate	274	23.3	457.14	304.95 168.87	Catechins	Olennikov et al. (2020), Zhang et al. (2022)
17	2-phenylethyl-vicianoside	277	23.7	461.18^b^	415.14 148.89	Phenylethanoids	Alperth et al. (2019), Avula et al. (2009), Han et al. (2016a)
18	Prodelphinidin trimer-tri-*O*-gallate	273	24.2	1,369.15 684.01^c^	1,200.15 1,031.10 913.11 608.04	Proanthocyanidins	−
19	Herbacetin-*O*-glucoside-*O*-glucuronide	270	24.9	639.04	477.01 463.05 301.01	Flavonols	Olennikov et al. (2020)
20	Herbacetin-*O*-glucoside-*O*- ramnoglucoside	275	25.3	771.14	609.05 463.04 447.08 301.05	Flavonols	Petsalo et al. (2006)
21	Rhodionidin (Herbacetin-7-*O*-rhamnoside-8-*O*-glucoside)	275	25.6	609.08	463.04 447.08 301.05	Flavonols	Petsalo et al. (2006)
22	Rhodioloside E	209	27.4	511.38	465.24 333.18 311.00 293.05	Monoterpene glycosides	Alperth et al. (2019)
23	Rosarin	250	28.3	473.19^b^	427.15 292.96	Phenylpropanoids	Alperth et al. (2019)
24	Rosavin^a^	250	28.9	473.21^b^	427.11 292.98	Phenylpropanoids	Alperth et al. (2019)
25	Rosiridin	200	29.6	377.19^b^	331.09 178.95	Monoterpene glycosides	Alperth et al. (2019)
26	Rosin	268	30.0	473.19^b^	427.10 293.07	Phenylpropanoids	Alperth et al. (2019)
27	Unknown	270	30.5	503.23^b^	457.12 293.02	−	−
28	Rhodiolgidin (Gossypetin-7-*O*-rhamnoside-8-*O*-glucoside)	274	35.1	625.34	479.06 316.96	Flavonols	Petsalo et al. (2006)
29	Sachaloside II/Sacranoside A	274	36.2	491.25^b^	445.19 313.00	Monoterpene glycosides	Iannuzzo et al. (2024)
30	Unknown	274	37.4	489.13^b^	443.14	−	−
31	Rhodiosin (Herbacetin-7-*O*-glucorhamnoside)	274, 330, 383	39.6	609.21	301.01	Flavonols	Olennikov et al. (2020)
32	Sachaloside II/Sacranoside A	212	40.3	491.18^b^	445.19 313.06	Monoterpene glucosides	Olennikov et al. (2020)
33	Rhodionin (Herbacetin-7-*O*-rhamnoside)	274, 385	40.8	447.52	301.22	Flavonols	Olennikov et al. (2020)
34	Rhodiooctanoside	213	43.4	469.20^b^	423.19 291.15	Acyclic alcohol glycosides	Iannuzzo et al. (2024)
35	Kenposide A	213	43.7	493.26^b^	447.29	Monoterpene glucosides	Olennikov et al. (2020)
36	Kaempferol	267, 360	53.0	285.30	(−)	Flavonols	Alperth et al. (2019)

a: Reference standard; b: Detected as [M + COOH]^−^ ; c: [M-2H]^2−^.

(−) No ionization in ESI-MS.

In the course of our analysis, we identified 36 metabolites, encompassing a diverse range of chemical groups, including phenolic acids, monoterpene alcohols glycosides, cyanogenic glycosides (hydroxynitrile glycosides), flavonoids, phenylethanoids, phenylpropanoids, proanthocyanidins, and others. Constituents were tentatively identified by comparison with literature data describing compounds previously found in this genus. Analysis was performed mainly considering negative ionisation data. Negative molecular ions are indicated by *m/z* values equal to [M−H]^−^ or [M−2H]^2−^. In several peaks, formic acid adducts were detected as ([M−H]^−^ + COOH), which fully dissociated into [M−H]^−^ in MS^2^.

The representative of the phenolic acid group was gallic acid (**1**, t_r_ = 4.3 min) with *m/z* 169.08 [M−H]^−^.

Compounds **2** and **3** were identified as hydroxynitrile glucosides structurally related to cyanogenic glycosides. Compound **2** (t_r_ = 6.5 min) showing a pseudomolecular ion at *m/z* 304.14 [M + COOH]^−^, with fragments at *m/z* 258.04 and 178.90 was identified as rhodiocyanoside A, while compound **3** was found to be lotaustralin (t_r_ = 9.9 min; *m/z* 260.08 [M−H]^−^).

Two phenyloethanoids were identified. Salidroside with *m/z* 299.02 [M−H]^−^ (**6**) is the most frequently reported phenyloethanoid, representing the *Rhodiola* genus, found in the aerial parts as well as roots as rhizomes (Zakharenko et al., 2021). Moreover, viridoside (**12**), identified at t_r_ = 17.9, with its pseudomolecular ion at *m/z* 359.31 [M + COOH]^−^ and fragmentation pattern at *m/z* 313.06 and 178.91 was also identified (Lee et al., 2016).

From the group of oligomeric procyanidins, compounds **11** (t_r_ = 16.7 min) and **15** (t_r_ = 21.9 min) were identified as prodelphinidin dimers, as its *O*–gallate (761.18 → 609.16 → 423.14 → 305.07) and di–*O*–gallate (913.19 → 761.18 → 608.76 → 423.14 → 305.07), respectively. Similarly, compound **18** (t_r_ = 24.2) was also classified into the proanthocyanidin group and identified as prodelphinidin trimer–tri–*O*–gallate (1,369.15 → 913.19 → 608.76).

Six glycosides of monoterpene alcohols were also identified (or partially identified) during the analysis. Compounds **7** and **22** were structurally elucidated and designated as rhodioloside D and rhodioloside E, respectively. High–resolution mass spectrometric analysis of compound **7** revealed a pseudomolecular ion at *m/z* 349.11 [M−H]^-^. Compound **22** was eluted at 22.4 min and exhibited a pseudomolecular ion at *m/z* 511.38 [M−H]^-^.

Rosiridin (rosiridol 1–*O*–glucoside), eluted at t_r_ = 29.6 min with *m/z* 331.09 [M−H]^−^ and fragmentation ion at *m/z* 178.95, corresponding to the loss of glucose moiety was identified as compound **25**. Sacranoside A and sachalinoside II can be seen as a possible candidates for **29** (t_r_ = 36.2) or/and **32** (t_r_ = 40.3) with their pseudomolecular ion at *m/z* 445.19 [M−H]^−^. Based on pseudomolecular ion at *m/z* 447.29 [M−H]^−^ compound **35** was identified as kenposide A.

A distinctive characteristic of *R. rosea* is the presence of cinnamic alcohol glucosides and a notably high concentration of phenylpropanoids, called rosavins, specified for *Rhodiola rosea*. Compounds **23** (t_r_ = 28.3 min), **24** (t_r_ = 28.9 min), and **26** (t_r_ = 30.0 min) were identified as rosarin, rosavin and rosin respectively, while rosavin was identified in comparison with reference substance. The sugar moieties consist of arabinopyranosyl–glucopyranoside for (**23**), arabinofuranosyl–glucopyranoside for (**24**), and glucopyranoside for (**26)** respectively.

Another important group of compounds identified in *R. rosea* roots/rhizomes are flavonols and their derivatives. Compound **31** (t_r_ = 39.6) with pseudomolecular ion *m/z* 609.21 [M−H]^−^ and fragmentation pattern *m/z* 445.17 [M−H−164]^−^ and 300.94 [M−H−146]^−^ corresponding to loss of glucose and rhamnose moiety was identified as rhodiosin (herbacetin–7–*O*–glucorhamnoside). Compound **37** (t_r_ = 40.8) was identified as rhodionin (herbacetin–7–*O*–rhamnoside), based on its fragmentation pattern with *m/z* 447.52 → 301.22 [M−H−146] ^−^. Compound **13** (t_r_ = 19.1), with pseudomolecular ion at *m/z* 755.29 [M−H]^−^, was identified as rhodiosine rhamnoside (herbacetin–3–*O*–glucorhamnoside–7–*O*–rhamnoside) based on its fragmentation pattern indicating the loss of 2 rhamnose and 1 glucose molecules. In turn, compound **28** (t_r_ = 35.1) was identified as rhodiolgidin (gossypetin–7–*O*–rhamnoside–8–*O*–glucoside), based on fragment ions including 316.96 [M−H−146–162]^−^, indicating the presence of aglycone–gossypetin. Compound **36** showed [M−H]^−^ ion at *m/z* 285.30 (t_r_ = 53.0 min) and could be identified as kaempferol.

### 3.3 LC–MS analysis of *Rhodiola rosea* roots dietary supplements

Phytochemical analysis and identification of the primary compounds were conducted on ethanolic extracts and infusions prepared from 13 dietary supplements declared to contain *R. roseae* roots.

Based on the comparison of the phytochemical profiles of various species within the *Rhodiola* genus, salidroside’s presence can be considered a generic marker compound. In addition to *R. rosea*, its presence has been recorded in several other species, including *R. quadrifida, R. algila* (Ledeb.) Fisch, *R. sachalinensis*, *R. kirilowii*, *R. crenulata*, *R. heterodonta*, and *R. semenovii* (Zakharenko et al., 2021). As demonstrated in our analysis, salidroside was confirmed in 11 out of the 13 tested formulations containing both *R. rosea* and other related species. Based on the peak sizes, the highest content of this compound was identified in K1, T3, and P1 samples. Notably, salidroside was not detected in T1/T1A and T2/T2A, while T2 did not contain any compounds characteristic of the *Rhodiola* genus, as confirmed by HPTLC and LC–MS methods.

On the other hand, the occurrence of cinnamic alcohol and its glycosides–rosavin, rosarin, and rosin, is described as a characteristic marker specific to the *R. rosea* species (Bykov et al., 1999). The confirmation of these marker compounds’ presence is a crucial factor in the quality assessment of formulations containing *R. rosea*. Based on the results obtained from HPTLC fingerprinting, the presence of rosavin was confirmed in only five samples (Z1, Z3, K1, K3, K5). However, using the more sensitive liquid chromatography–mass spectrometry (LC–MS) method, rosavin was additionally detected in trace amounts in three further samples (Z2, K2, K4). This may indicate the poor quality of certain preparations, including the predominant presence of contaminants or a significant presence of other *Rhodiola* species in their composition.

In turn, in 5 of the tested samples, all three phenylpropanoids (**rosarin, rosavin, rosin**) were identified. In samples **K4** and **Z2, Z4** a small (trace) amount of rosavin was possibly detected using LC–MS, along with compounds characteristic of other *Rhodiola* species, such as *R. crenulata*, *R. gelida*, *R. sacra*, and others, e.g., crenulatin and creoside I.

The characteristics of the individual forms of administration of the tested dietary supplements declaring the content of *R. rosea* or their extracts are presented below. The detailed characteristics of the presence of compounds identified in the individual samples are summarised in Table 3, available below. The numbering of the compounds in the graphical representations corresponds to that in Table 3.

**TABLE 3:** The content of compounds in individual dietary supplements identified on the basis of HPLC-MS - retention times, UV, mass spectra fragmentation.

No	Compound	UV [nm]	Rt [min]	[M-H]^-^	Ref material	Z1/Z1A	Z2/Z2A	Z3/Z3A	Z4/Z4A	K1/K1A	K2/K2A	K3/K3A	K4/K4A	K5/K5A	P1/P1A	T1/T1A	T2/T2A	T3/T3A
1	Gallic acid	271	4.3	169.08	+	+	+	+	+	+	+	+	+	+	+	+	-	+/−
2	Rhodiocyanoside A	208	6.5	304.14^b^	+	+	+	+	+	+	+	+	+	+	+	+	-	-
3	Protocatechuic acid-*O*-glucoside	254, 280	8.7	315.27	-	-	-	-	-	+/−	-	-	-	-	+	+	-	+
4	Lotaustralin	203	9.9	306.11^b^	+	+	+	+	+	-	+	+	-	+/−	+	+	-	-
5	Unknown	205	11.6	306.16	+	+/−	-	-	+/−	-	-	-	-	-	-	-	-	-
6	Crenulatin (I)	190	12.0	293.17	-	-	-	-	-	+	-	-	+	-	+/−	-	-	+
7	Unknown	267	12.3	343.11	+	-	-	-	-	-	-	-	-	-	-	-	-	-
8	Salidroside	215, 280	12.8	345.22^b^	+	+	+	+	+/−	+	+	+	+	+	+	-	+	+
9	*p*-Coumaric acid glucoside	205	12.9	371.58^b^	-	-	+	-	+	-	-	-	-	-	-	-	-	-
10	Rhodioloside D	208	13.4	395.22^b^	+	+	-	+	-	-	+	+	-	+	-	-	-	-
11	Unknown	207	14.5	293.16	+	+	+	+	+/−	-	-	-	-	+/−	+	-	-	-
12	Crenulatin (II)	195	14.8	293.16	-	-	-	-	-	+	-	-	+	+/−	-	-	-	-
13	Epigallocatechin	275	15.1	305.16	+	+	+	+/−	+	-	-	-	-	+	-	+	-	-
14	Salidroside pentoside	275	15.8	431.29	-	-	-	-	-	-	-	-	-	-	+	-	-	+
15	Olivil-*O*-glucoside	207	16.2	537.16	+	+	-	-	+	-	-	-	-	-	+/−	-	--	-
16	Prodelphinidin B-2 3-*O*-gallate	209	16.7	761.18	+	+	+	+	+	+	+/−	+	+/−	+	+	+	-	-
17	Creoside I	203	16.8	349.00^b^	-	-	-	-	-	+	-	-	+	-	-	-	-	-
18	Viridoside	263	17.9/18.1	359.31^b^	+	-	-	-	-	-	-	+	-	-	-	-	-	-
19	Herbacetin-3-*O*-glucorhamnoside−7-*O*- rhamnoside	202, 275	19.1	755.29	+	+	+	+	-	-	+	+	-	+	-	-	-	-
20	Benzyl alcohol arabinosyl- glucoside	209	19.3	447.37^b^	+	+	+/−	+/−	+/−	+	-	-	-	+/−	+/−	+	-	-
21	Creoside II	209	19.8	351.48^b^	-	-	-	-	-	+/−	-	-	+/−	-	-	-	-	-
22	Unknown	216	20.0	403.24	-	-	-	-	-	-	-	-	-	-	-	-	-	+
23	Proantocyjanidin dimer	210	20.8	577.45	-	-	+	-	-	+	-	-	+	-	-	-	-	-
24	Unknown	210	21.2	415.13	-	-	-	-	-	+	-	-	+/−	-	-	-	-	+
25	Prodelphinidin B-2 3,3′-di-*O*-gallate	208, 275	21.9	913.16	+	+	+	+	+	-	+	+	-	+	+	+	-	-
26	Unknown	213	22.2	445.28	-	-	-	-	-	-	-	-	-	-	-	-	-	+
27	Unknown	208	22.4	761.24	-	+	-	+/−	+	-	-	-	-	-	+	+	-	-
28	Epigallocatechin gallate	206, 274	23.3	457.14	+	+	+	+	+	+	+	+	-	+	+	+	-	-
29	2-phenylethyl-vicianoside	207	23.7	461.18^b^	+	+	+	+	+	-	+	+	-	+	+	+	-	-
30	Prodelphinidin trimer-tri-*O*-gallate	273	24.2	1,369.15 684.01^c^	+	+	+	+	+	-	+/−	+	-	+	+/−	+	-	-
31	Herbacetin-O-glucoside-*O*-glucuronide	270	24.9	639.04	+	-	-	-	-	+	-	-	-	-	-	-	-	-
32	6′-*O*-galloylsalidroside	208	25.1	451.51	-	+	-	+/−	+	+	+/−	+	+	+	+	-	-	-
33	Herbacetin-O-glucoside-*O*- rhamnoglucoside	275	25.3	771.14	+	+	-	+	-	+/−	+/−	-	+/−	+/−	-	-	-	-
34	Rhodionidin (Herbacetin-7-*O*-rhamnoside-8-*O*-glucoside)	275	25.6	609.08	+	+	-	+	-	+	+	+	+	+	+/−	-	-	+
35	Trigalloyl glucose (TGG)	217	25.6	787.14	-	-	-	-	-	-	-	-	+	-	-	-	-	-
36	Unknown (Phenylethanoid)	330	26.0	785.30	-	-	-	-	-	+	-	-	-	-	-	-	-	+
37	Unknown	209	27.4	511.38	+	+	+	+	+	+	+	+	+	+	+	+	-	-
38	Rosarin	250	28.3	473.21^b^	+	+	-	+	+/−	+/−	+/−	+	+/−	+	-	-	-	-
39	Creoside IV	212	28.9	441.30^b^	-	-	-	-	-	-	-	-	-	-	+	+	-	-
40	Rosavin	250	28.9	473.21^a,b^	+	+	+/−	+	+/−	+/−	+/−	+	+/−	+	-	-	-	-
41	Rosiridin	200	29.6	377.19^b^	+	+	+/−	+	+	+	+	+	+	+	-	-	-	-
42	Prodelphinidin B-di-*O*-gallate	210	29.9	913.13	-	-	+	-	+	-	-	-	-	-	+	+	-	-
43	Trigalloyl glucose (TGG) (II)	217	29.9	787.16	-	-	-	-	-	-	-	-	+	-	-	-	-	+
44	Rosin	268	30.0	473.19^b^	+	+	-	+	-	-	+/−	+	+/−	+	-	-	-	-
45	Unknown	216	30.1	569.23	-	-	-	-	-	+/−	-	-	-	-	-	-	-	+
46	Unknown	273	30.5	503.23^b^	+	+	-	+	-	-	-	-	-	-	-	-	-	-
47	Proanthocyanidin	270	31.3	684.12	+/−	+	+	+	+	-	+/−	+	-	+/−	+	+	-	+
48	Unknown (Phenylethanoid)	325	33.4	623.29	-	-	-	-	-	+	-	-	-	-	+/−	-	-	+
49	Pentagalloylo glucose (PGG)	217, 279	33.5	939.12	-	-	+/−	+/−	-	-	+/−	+/−	+	+/−	+/−	-	-	+
50	Rhodiolgidin (Gossypetin-7-*O*-rhamnoside-8-*O*-glucoside	274	35.1	625.34	+	+/−	-	+/−	-	-	-	-	-	+	+/−	-	-	-
51	Sachaloside II/Sacranoside A	274	36.2	491.25^b^	+	+	+	+	+/−	-	+	+	-	+	+/−	+/−	-	-
52	Unknown	274	37.4	489.13^b^	+	+	+/−	+	-	-	-	-	-	-	-	-	-	-
53	Rhodiosin (Herbacetin-7-*O*-glucorhamnoside)	274, 330	39.6	609.21	+	+	-	+	-	+	+	+	+	+	+/−	+/−	-	-
54	Sachaloside II/Sacranoside A	212	40.3	491.18^b^	+	+	+	+	+	-	-	+	-	+	-	+	-	-
55	Rhodionin (Herbacetin-7-*O*-rhamnoside)	274	40.8	447.52	+/−	+/−	-	+	-	+	+	+	+	+	+/−	-	-	-
56	Rhodiooctanoside	213	43.4	469.20^b^	+	+	+/−	-	+	+	+	-	+	+	+	+	-	+/−
57	Kenposide A	213	43.7	493.26^b^	+	+	+	+	+	+	+	+	+	+	+	+	-	+
58	Kaempferol	221	53.0	285.30	+	+	-	-	-	-	-	-	+	+/−	-	-	-	-
59	Feruloyl hexanoic acid	221, 322	54.9	307.34	-	+	+	-	+	-	-	-	-	-	+	+	-	-

a: Compared with reference standard; b: Detected as [M + COOH]^−^; c: [M-2H]^2−^; +/−: trace.

#### 3.3.1 Cut raw material

**FIGURE 3:**
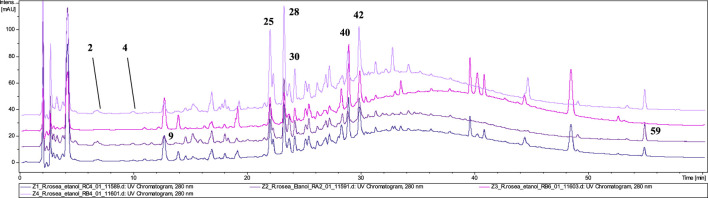
LC-DAD-MS/MS chromatograms of 60% ethanolic extracts prepared from tested cut raw materials*,* recorded at 280 nm.

The phytochemical analysis of the preparations in the form of unprocessed raw material confirmed the presence of rosavin (**40**, t_r_ = 28.9) as a marker compound for *R. rosea* in four tested preparations (**Z1–Z4**) (Figure 3). However, in samples **Z2** and **Z4**, the presence of rosavin was minimal. However, the presence of compounds of ambiguous species affiliation was observed. In both **Z2** and **Z4** *p*–coumaric acid glucoside (**9**) with *m/z* 371.25 [M + COOH]^−^ (MS^2^ 325.05 and 162.84 [M−H]^−^) and feruloyl hexanoic acid (**59**) with *m/z* 307.34 [M−H]^−^ (MS^2^ 193.00 [M−H]^−^) characteristic for *R. wallichiana*, were detected (Liu et al., 2019). Phytochemical profile of **Z4** was matching with our reference extract of *R. kirilowii* (data nor shown), especially rich in proanthocyanidins such as: prodelphinidin B–2 3,3′–di–*O*–gallate (**25**), epigallocatechin gallate (**28**), prodelphinidin trimer–tri–*O*–gallate (**30**) and prodelphinidin B–di–*O*–gallate (**42**). This may suggest the presence of a mixture of species in the tested preparations (*R. rosea*, *R. wallichiana* and *R. kirilowii*), although we did not identify the presence of compounds typical of *R. crenulata*, e.g., crenulatin or creosides I–IV. Based on the HPTLC chromatogram and comparison of the LC–MS **Z1** and **Z3** fingerprints with the reference material profile, we can determine the presence of the *R. rosea* species as the leading (or only) component.

#### 3.3.2 Capsules

**FIGURE 4:**
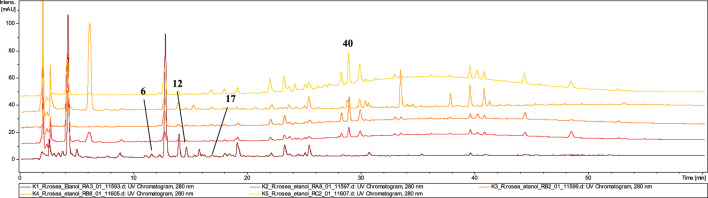
LC-DAD-MS/MS chromatograms of 60% ethanolic extracts prepared from tested capsules*,* recorded at 280 nm.

The analysis of the capsules’ composition confirmed the presence of rosavin (**40**, t_r_ = 29.0) in all five tested extracts (K1–K5) (Figure 4). However, the content of this compound in formulations **K1** and **K4** was only in trace amount. Interestingly, despite the presence of this compound characteristic of *R. rosea*, the identity of this species could only be conclusively confirmed in 2 of the 5 tested preparations–**K3**, and **K5**. In capsules K1 and K4, only minimal amounts of rosavins were detected, accompanied by the presence of compounds characteristic of other *Rhodiola* species. Notably, creoside I (**17**, t_r_ = 16.8) with *m/z* at 349.00 [M + COOH]^-^ and crenulatin (**6**, t_r_ = 12.0 and **12**, t_r_ = 14.8), which are also characteristic of *R. crenulata*, were identified (Han et al., 2016a; Han et al., 2016b).

#### 3.3.3 Powder

**FIGURE 5:**
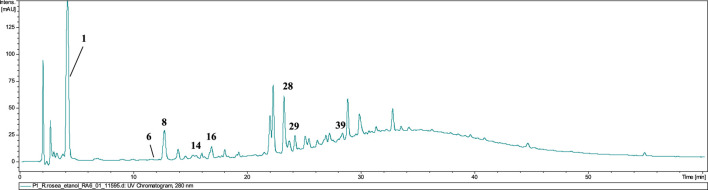
LC-DAD-MS/MS chromatograms of 60% ethanolic extract prepared from tested powder*,* recorded at 280 nm.

The dominating compounds of P1 powdered raw material were gallic acid (**1**, t_r_ = 4.3), identified by its pseudomolecular ion at *m/z* 169.08 [M−H]^−^, salidroside (**8**), salidroside pentoside (**14**), prodelphinidin B–2 3–*O*–gallate (**16**), epigallocatechin gallate (**28**), and 2–phenylethyl–vicianoside (**29**). No rosavin was detected in the P1 extracts, confirming the absence of *R. rosea* (Figure 5). Additionally, the presence of traces of crenulatin (**6**) and creoside IV (**39**, t_r_ = 28.9) in the tested extracts suggests potential adulteration of the preparation with *R. crenulata* with other undefined *Rhodiola* *spp.* excluding *R. rosea* (Han et al., 2016a; Han et al., 2016b).

#### 3.3.4 Tablets

**FIGURE 6:**
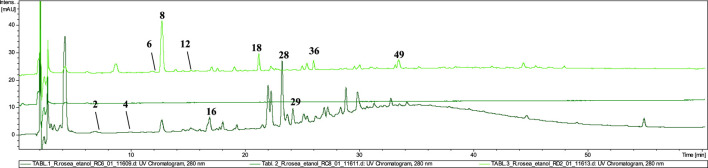
LC-DAD-MS/MS chromatograms of 60% ethanolic extracts prepared from tested tablets*,* recorded at 280 nm.

The dietary supplements in **tablet** form (T1, T2, T3) exhibited the lowest quality among the tested preparations (Figure 6). LC–MS analysis revealed that none of the three tablet–formed preparations contained extracts from *R. rosea* roots/rhizomes. Neither rosavin nor other characteristic cinnamic alcohol derivatives were identified in **T2** and **T3** extracts.

However, in extract **T1**, we identified compounds characteristic of other species within the *Rhodiola* genus, such as prodelphinidin B–2 3–*O*–gallate (**16**), epigallocatechin gallate (**28**), and 2–phenylethyl–vicianoside (**29**), which are also commonly found in *R. gelida*, *R. recticaulis*, *R. sachalinensis*, *R. kirilowii* and *R. sacra* (Tao et al., 2019). In tablet **T3**, compounds specific to *R. crenulata*, such as crenulatin (**6**, t_r_ = 12.0 and **12**, t_r_ = 14.8) with *m/z* at 293.17 [M−H]^−^ was detected together with compound not present in *Rhodiola spp*. (compounds **36** and **49**) (Nakamura et al., 2008). Moreover, similarly to the P1 preparation, in the T3 tablet we observed the dominant presence of salidroside (**8**) and small amounts of its derivative–salidroside pentoside (**14**).

### 3.4 Anti–inflammatory activity of *Rhodiola rosea* root extracts

Microglial cells represent the primary immune defense within the central nervous system (CNS), playing a crucial role in responding to pathological stimuli and maintaining neural homeostasis. To assess the biological activity of the tested samples (towards TNF-α and IL-6 release), we employed the LPS-stimulated BV2 microglial cell line, a widely used *in vitro* model for investigating the effects of plant-derived compounds on neuroinflammatory processes (Marrazzo et al., 2023).

**FIGURE 7:**
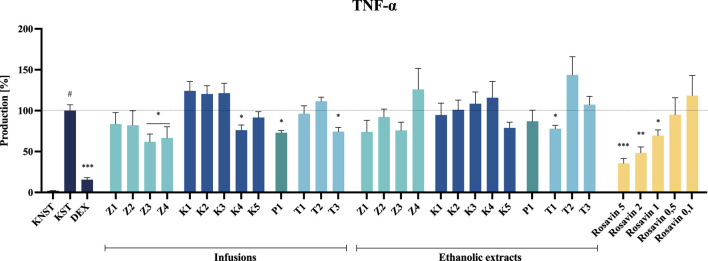
The influence of tested extracts (50 μg/mL) and rosavin (0.1–5 μM) on TNF-α secretion by LPS-stimulated microglia BV2. Data from three separate experiments assayed in duplicate are expressed as mean ± SEM. Dexamethasone (DEX, 20 μM) was used as a positive control. Absorbance values for all samples were expressed as percentages relative to the LPS-stimulated control (KST), which was set at 100%. Statistical significance: *p < 0.05, **p < 0.01, ***p < 0.001 vs. stimulated control (KST), # statistically significant (*p* < 0.001) vs. non-stimulated control (KNST).

The most substantial inhibition of TNF-α secretion was observed following incubation with infusions derived from the Z3 raw material, which contained the highest concentration of rosavin among the unprocessed samples, as indicated in the Supplementary Material (Supplementary Figure S3). Furthermore, infusions prepared from formulations containing only trace amounts or entirely lacking the marker compound, including Z4, capsule K4, tablet T3, powder P1, and the T1 ethanolic extract, also demonstrated inhibitory effects. For example, ethanolic extracts from products Z3 and Z4 reduced TNF-α secretion to 62.0% ± 9.5% and 66.6% ± 13.7%, respectively. In contrast, incubation of cells with K4, T3 ethanolic extracts, and the T1 infusion induced TNF-α secretion at levels of 76.0% ± 6.5%, 74.3% ± 5.1%, and 77.8% ± 4.3%, respectively, relative to the LPS–stimulated control (100% secretion) (Figure 7).

Despite the noticeable inhibitory effect of certain tested preparations (e.g., ethanolic extracts from raw materials or the infusion from P1) on IL-6 secretion, none of the analyzed samples demonstrated a statistically significant effect at the tested concentration.

**FIGURE 8:**
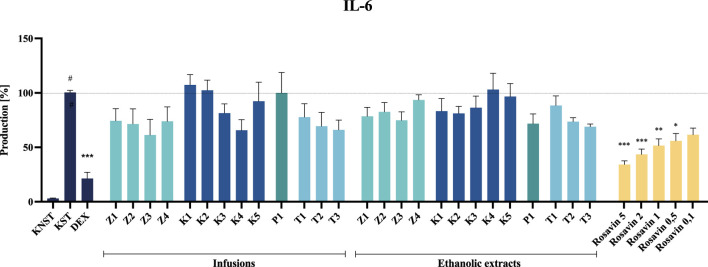
The influence of tested extracts (50 μg/mL) and rosavin (0.1–5 μM) on IL-6 secretion by LPS-stimulated microglia BV2. Data from three separate experiments assayed in duplicate are expressed as mean ± SEM. Dexamethasone (DEX, 20 μM) was used as a positive control. Absorbance values for all samples were expressed as percentages relative to the LPS-stimulated control (KST), which was set at 100%. Statistical significance: *p < 0.05, **p < 0.01, ***p < 0.001 vs. stimulated control (KST), # statistically significant (*p* < 0.001) vs. non-stimulated control (KNST).

Rosavin, as the primary marker compound, exhibited a statistically significant, concentration–dependent inhibitory effect on the secretion of the tested inflammatory mediators. Notably, rosavin suppressed TNF-α secretion at concentrations of 5, 2 and 1 μM, reducing its levels to 35.5% ± 6.0%, 48.5% ± 6.9% and 69.5% ± 6.9% respectively, relative to the LPS-stimulated control (100% secretion) (Figure 7). The effect of rosavin on IL-6 secretion was equally pronounced. The analyzed compound significantly reduced IL-6 levels to 34.3% ± 3.3% at a concentration of 5 μM, 43.5% ± 4.9% at 2 μM, and 51.6% ± 6.2% at 1 µM (Figure 8).

Extracts prepared from dietary supplements of questionable quality or those containing significant admixtures of other species exhibited minimal or no effect on LPS-stimulated mediator production in the BV2 cell model at a concentration of 50 μg/mL (Figures 7, 8). None of the tested samples exhibited cytotoxic effects on BV2 microglial cells (see Supplementary Material, Supplementary Figures S4, S5).

## 4 Discussion

Neuroinflammation plays a crucial role in the pathogenesis of neuropsychiatric and neurodegenerative disorders. A growing body of evidence suggests that excessive microglial activation and the overproduction of pro–inflammatory mediators underlie the development of psychophysical exhaustion, depression, anxiety, and neurodegeneration. In addition to its role in synaptic plasticity, learning, and memory, microglia are essential for maintaining central nervous system (CNS) homeostasis. Due to their involvement in these processes, microglia may become an important target for therapeutic interventions aimed at modulating neuroinflammatory responses and mitigating disease progression (Gao et al., 2023; Li et al., 2022).

*Rhodiola rosea* L., a traditional medicinal plant widely used in Eastern Europe and Asia, is recognized for its neurostimulatory, antidepressant, and adaptogenic properties. Studies have demonstrated its efficacy in enhancing cognitive performance, reducing fatigue, and alleviating stress–related symptoms. In shift workers, short–term administration of *R. rosea* extract improved concentration, focus, and reaction speed under stress. Similarly, in a study on 80 healthy students, a 14–days regimen of 400 mg *R rosea* root extract significantly reduced anxiety, stress, anger, and confusion while improving mood (Cropley et al., 2015). Additionally, a phase II clinical trial comparing *R. rosea* with sertraline indicated a moderate antidepressant effect in mild to moderate depression, with fewer adverse effects than the conventional drug (Mao et al., 2015). The available literature provides partial insights into the pathomechanism underlying this effect. Available review reports systematize the available evidence indicating the inhibitory effects of the raw material on the secretion of inflammatory mediators, reactive oxygen species (ROS), and nitric oxide (NO) as well as its anti–apoptotic functions in *in vitro* models employing microglial and macrophage cells. Notably, the majority of studies focus on assessing the bioactivity of isolated single compounds, predominantly salidroside, a constituent found in multiple species within this genus and rosavins as leading anti–inflammatory components (Liu et al., 2024). Furthermore, there is a limited number of studies that establish a correlation between the biological activity of the extract and its chemical composition (Ma et al., 2018; Pu et al., 2020).

The growing global demand for *Rhodiola rosea* products, spurred by increasing scientific evidence supporting its efficacy, presents significant challenges to the conservation and sustainable use of this species. The expanding use of *R. rosea* in herbal medicines, dietary supplements, cosmetics, and food additives, combined with its limited cultivation areas, high agricultural costs, lengthy growing period, low dispersal ability, and low rate of germination and seedling survival is contributing to a gradual decline in the availability of this raw material worldwide (Galambosi, 2006). Depletion of natural populations and habitat destruction have led to the disappearance of *Rhodiola* species in many regions, as most of the raw material is collected from the wild, and the plant needs several years to regenerate (Howes et al., 2020).

The increasing demand for raw materials, coupled with their decreasing availability, has led to a significant rise in cases of intentional and accidental adulteration of *R. rosea* products. A major concern in this context is the admixture or substitution of *R. rosea* roots and rhizomes with those from other *Rhodiola* species, which are morphologically similar and difficult to distinguish, particularly in underground structures. Recent phylogenetic studies have highlighted significant evolutionary convergence among the 74 species of *Rhodiola* (Zhang et al., 2014). Although the color of the flowers can differentiate *R. rosea* from *R. crenulata* and other purple–flowering species, this convergence is particularly noticeable in the roots and rhizomes of *Rhodiola*, which lack distinct diagnostic features, making species identification based on these structures difficult (Cuerrier A and Rapinski, 2014; Zhu et al., 2017).

According to a 2016 market study, approximately 75% of the global *Rhodiola* extract production originates from Asia, with around 13% sourced from Europe, 5% from the United States, and 7% from other regions (Center, 2016). The high volume of imports from Asia, particularly from China, into the U.S. and European herbal supplement markets has led to frequent adulteration or substitution of *R. rosea* raw materials with other Asian *Rhodiola* species. This is compounded by the interchangeable use of various *Rhodiola* species names in different regions, with species like *R. crenulata*, *R. heterodonta*, and *R. kirilowii* being labeled under the common term “rhodiola” in China (Cunningham et al., 2020). Despite limited data, it is widely recognized that European sources of *R. rosea* are scarce. The commercial collection of *R. rosea* in Europe is of relatively low economic significance due to high labor costs and logistical challenges associated with harvesting in remote mountainous regions. Furthermore, many European populations of *R. rosea* are legally protected, including those in Bulgaria, the Czech Republic, Austria, and Germany (Fisheries, 2022).

The United States Herbal Medicines Compendium identifies several *Rhodiola* species as potentially confounding in the commercial supply of *Rhodiola rosea*, including *R. crenulata*, *R. kirilowii*, *R. sacra*, *R. serrata*, *R. sachalinensis*, and *R. yunnanensis* (United States Pharmacopeial Convention, 2014). Our analysis indicates the intentional or accidental substitution of *R. rosea* with *R. crenulata* in at least 5 of the tested products, as suggested by the presence of compounds characteristic of *R. crenulata*, such as creoside I, IV, and crenulatin. A typical example of a species involved in the adulteration of roseroot preparations is also *R. sachalinensis*, a species with a similar chemical composition to *R. rosea* but containing lower amounts of rosavins and salidroside. Some botanists even regard *R. sachalinensis* as the same species or a subspecies of *R. rosea* (Gontcharova et al., 2009). However, chemotaxonomic studies conducted by Booker et al. show differences between *R. rosea* and *R. sachalinensis*, which, based on general metabolomic methods and specific high–performance thin–layer chromatography (HPTLC) spectra and limited geographical range, allow treating them as separate species (Booker et al., 2016; Liu et al., 2013).

Although adulteration of preparations with other *Rhodiola* species seems to be a major challenge for the pharmaceutical market, meeting the requirements for good quality raw materials for the growing industrial demand is also a significant problem. The key aspect seems to be the requirements of the European Pharmacopoeia for *R. rosea* roots and rhizomes in terms of the content of marker compounds, i.e., salidroside and rosavins.

*R. rosea* is available on the market in various forms, including cut/dried, powdered rhizomes and roots, dry or liquid extracts, as well as dry extracts in solid dosage forms like capsules and tablets (Brinckmann et al., 2021). Market analysis indicates that the global demand for *R. rosea* products is constantly growing, and with it, the number of adulterated or poor–quality preparations (Bernard, 2016). The scale of the problem is illustrated by earlier studies assessing the quality of preparations declaring the content of *R. rosea,* available on the European market. Ma et al. found that approximately 33% of 18 commercial *Rhodiola rosea* rhizome powder extract samples did not display a consistent Rapid Resolution Liquid Chromatography (RRLC) profile and were missing the characteristic peaks of rosarin, rosavin, and rosin or did not meet the claimed contents standards for the marker compounds for authentic *R. rosea* rhizomes (Ma et al., 2011). Similarly, a study by Booker A. et al. (2016) assessing the composition of 40 commercial preparations available on the EU market, claiming to contain *R. rosea*, confirmed the absence of rosavin in approximately 20% of the cases. Furthermore, in the remaining 80%, based on NMR Spectroscopy and HPTLC methods, the rosavin content was lower than declared. Additionally, 25% of tested dietary supplements were found to be substituted with other *Rhodiola* species, and one product was adulterated with synthetic 5–hydroxytryptophan (5–HTP) (A. Booker et al., 2016). A study conducted by Ruhsam M et al., in 2018 found that only 5 of 13 roseroot supplements contained *R. rosea*, with no visible admixture of other species (Ruhsam and Hollingsworth, 2018). In a study presented by Marchev A. et al., of 30 commercial products purchased from various suppliers, only 23% had rosavin and salidroside levels consistent with the amounts specified on the label, as confirmed by HPLC analysis. Additionally, 37% of the products did not meet the typical standardisation (1% salidroside and 3% rosavins), which may suggest potential adulteration (Marchev et al., 2020). In the study by Cunningham A. et al., they also found the presence of additives of extracts from the roots of *Astragalus* and *Lycium* species in preparations, declaring the presence of species of the *Rhodiola* genus (Cunningham et al., 2020).

Despite a smaller research group, our study indicates that almost 60% of the tested preparations did not contain the declared content of a single raw material–*R. rosea* roots/rhizomes or their extracts. Moreover, we detected the presence of rosavin in only 9 out of 13 tested preparations (using the LC–MS method), with 4 of these showing only trace amounts of the marker compound. Notably, the misidentification or accidental use of other *Rhodiola* species was most frequently observed in the tablet/capsule group. This could be attributed to the ease of substituting powdered extracts and the challenges associated with their precise identification.

Numerous studies have evaluated the quality and composition of commercial *R. rosea* products, highlighting the potential substitution of *R. rosea* with other *Rhodiola* species in dietary supplements. Such substitutions are often driven by the greater availability and ease of large–scale cultivation of alternative species. Notably, while the substitution of *R. rosea* with *R. crenulata* is widely recognized as a primary authenticity issue in *R. rosea* products, *R. crenulata* roots and rhizomes are preferred in Traditional Chinese Medicine (TCM) due to their higher availability in Asia, consistent with previous reports. However, from a safety perspective, the consumption of *R. rosea* is endorsed by the European Medicines Agency, whereas other species within the *Rhodiola* genus lack similar regulatory support. Furthermore, prior research underscores the efficacy of *R. rosea* as an adaptogen, demonstrating its beneficial effects in managing stress–related conditions, enhancing both mental and physical performance, and supporting immune function. In addition, few reports indicate its potential effectiveness in the adjunctive therapy and prevention of microglia–generated neuroinflammation associated with the development of neurodegenerative diseases, e.g., Parkinson’s or Alzheimer’s disease (Morgan and Grundmann, 2017).

The interchangeable use of different species within the same genus may, therefore, create some variations in chemical composition, potentially affecting the quality, safety, and biological activity of the product. The assessment of the anti–inflammatory effect of the tested preparations in the model of LPS–stimulated microglia may allow for the indirect correlation of the quality of the tested extracts with their biological effect.

Among the tested samples, the highest anti–inflammatory activity was observed for Z3 and Z4 among the unmodified raw material samples, K4 among the capsule formulations, T1 and T3 among the tablet formulations, and P1 in the powdered form (Figures 7, 8). Notably, the most significant inhibition of both TNF–α and IL–6 secretion was recorded for the ethanolic extract of preparation Z3, which was confirmed to contain *Rhodiola rosea*. Specifically, the infusion of Z3 resulted in TNF–α secretion inhibition, reducing its level to 62.0% ± 9.5%. Furthermore, Z3 reduced IL–6 secretion to 61.4% ± 14.2% and 74.8% ± 7.7% for the ethanol extract and infusion, respectively, relative to the 100% release observed in the stimulated control (Figure 8). The anti–inflammatory effects of *R. rosea* crude extract and its primary constituents, as well as their concentration–dependent modulation of iNOS, TNF–α, IL–1β, IL–6, and other inflammatory mediators, have been previously documented in the studies of Lee et al. and Jiang et al., among others (Borgonetti et al., 2020; Jiang et al., 2024; Lee et al., 2013). These findings are particularly relevant in the context of neuroinflammation modulation, a mechanism proposed to underlie the anti–stress effects of adaptogenic formulations (Ahmed et al., 2016; Guo et al., 2021).

An analysis of the composition of biologically active preparations suggests that their anti–inflammatory effects may partially correlate with the concentration of the marker compound for *Rhodiola rosea*. A comparative assessment of rosavin peak intensities in the analyzed samples of unprocessed plant material (Supplementary Table S3A, Supplementary Material) revealed a significantly higher concentration of rosavin in the Z3 preparation compared to the other samples.

Previous research has emphasized the role of phenylpropanoid derivatives, such as rosavins, and phenylethanol derivatives in contributing to the adaptogenic properties of *R. rosea*, exerting pleiotropic pharmacological effects on the neuroendocrine and immune systems (Fan et al., 2022; Maadawi, 2017). However, most studies have primarily focused on the biological activity of salidroside, with limited investigations into the effects of other compounds, including rosavin, within this cellular model (Lee et al., 2013; Zhang et al., 2022).

Thus, the neuroinflammation model employed in the present study enabled the evaluation of rosavin activity, representing one of the first investigations of this compound in a microglial cell model. As demonstrated, rosavin significantly and dose–dependently inhibited IL–6 and TNF–α secretion in LPS–stimulated microglia. Specifically, at a concentration of 5 μM, rosavin reduced IL–6 secretion to 34.3% ± 3.3% compared to the stimulated control (100% secretion), while a 43.5% ± 4.9% inhibition was observed at a concentration of 2 µM (Figure 8). Although data on rosavin’s effects in microglial cell models remain scarce, the present findings align with previous reports on its anti–inflammatory properties in other models, such as murine macrophages (Liu et al., 2024).

Interestingly, some preparations that did not contain *R. rosea* or rosavin—specifically P1, T1, and T3—also exhibited notable anti-inflammatory activity. These findings indicate that, although the selected cellular model is effective in capturing the activity of rosavin and standardized extracts, it may be insufficient for evaluating the overall quality or authenticity of complex commercial formulations.

It is also important to note that, although the BV2 microglial cell line is an immortalized murine model that does not fully capture the complexity of human microglia, it remains a widely used and well-established system for the preliminary investigation of neuroinflammatory mechanisms. Owing to their reproducible and well-characterized response to pro-inflammatory stimuli such as LPS, BV2 cells offer a practical and informative platform for the initial evaluation of anti-inflammatory properties in natural products (Marrazzo et al., 2023). Nevertheless, limitations related to species origin and monoculture conditions highlight the need for future studies employing more physiologically relevant models, including human-derived systems or co-culture approaches, to more accurately assess the biological activity of such preparations.

Nevertheless, our study confirms the potential of *Rhodiola rosea* as an agent mitigating neuroinflammatory processes, with rosavin identified as a compound potentially responsible for the observed biological activity of the raw material. With regard to other species within the *Rhodiola* genus, a comprehensive analysis should be conducted to assess their biological activities and to identify the specific compounds responsible for the observed effects.

The growth of the dietary supplement market necessitates detailed analyses of product composition and quality control. The increasing number of companies producing dietary supplements, along with rising consumer demand increases the risk of intentional or accidental contamination or incorrect product composition. European regulations governing the content of medicinal products containing *Rhodiola rosea* roots, rhizomes or their extracts necessitate the production of high–quality preparations with confirmed efficacy. The questionable quality of available preparations, claiming to contain *R. rosea* roots or rhizomes, may stem from either intentional or unintentional substitution of raw materials, as well as improper storage, which can lead to the degradation of key marker compounds. The objectives of this study were achieved by using an interdisciplinary approach (primarily based on LC–MS analysis) to better understand the sources of potential adulteration of individual herbal samples and their impact on biological activity. The identification of *R. rosea* in only 40% of the preparations underlines that rigorous control and standardisation of herbal supplements are crucial to understanding their therapeutic activity and preventing adulteration.

## Data Availability

The original contributions presented in the study are included in the article/Supplementary Material, further inquiries can be directed to the corresponding author.
