# Supplementary material for: Comprehensive profiling of Rhodiola rosea roots and corresponding products: phytochemical insights and modulation of neuroinflammation in BV2 microglial cell model
Source: Front Pharmacol. 2025 Jul 2;16:1608767. doi: 10.3389/fphar.2025.1608767 (PMC12264437; doi:10.3389/fphar.2025.1608767)
Supplement: Supplementary file 1 [file Supplementaryfile1.docx]

Supplementary Material

# Supplementary Figures and Tables

**Table S1:** Detailed information about tested products.

| **Sample ID** | **Declaration (species)** | **Origin of the product (country)** | **Origin of the plant material** | **Sample type** | **Source** | **Form** | **Standardization** |
| --- | --- | --- | --- | --- | --- | --- | --- |
| K1 | *Rhodiola rosea* root | Poland | Not given | Dietary supplement | Pure Herbs | Capsules | Extract standardised for 4% salidroside |
| K2 | *Rhodiola rosea* root | Poland | Not given | Dietary supplement | Swanson | Capsules | - |
| K3 | *Rhodiola rosea* root | Poland | Not given | Dietary supplement | Activlab | Capsules | Extract standardised for 3% salidroside |
| K4 | *Rhodiola rosea* root | Poland | Not given | Dietary supplement | Pharmovit | Capsules | Extract 4:1 |
| K5 | *Rhodiola rosea* root | Poland | Not given | Dietary supplement | Pharmovit | Capsules | Extract standardised for 3% rosavin and 1% salidroside |
| T1 | *Rhodiola rosea* root | Poland | Not given | Dietary supplement | Alter medica laboratories | Tablets | Powdered roots, standardised to 3,2% rosavin and 1% salidroside |
| T2 | *Rhodiola rosea* root | Poland | Not given | Dietary supplement | Herbapol | Tablets | Extract 4:1 |
| T3 | *Rhodiola rosea* root | Poland | Not given | Dietary supplement | My life | Tablets | Extract standardised for 3% salidroside |
| Z1 | *Rhodiola rosea* root | Poland | Russia | Dietary supplement | Natura wita | Cut raw material | - |
| Z2 | *Rhodiola rosea* rhizome | Poland | Poland | Dietary supplement | Dary natury | Cut raw material | - |
| Z3 | *Rhodiola rosea* root | Poland | Russia | Dietary supplement | Nanga | Cut raw material | - |
| Z4 | *Rhodiola rosea* root | Poland | Russia | Dietary supplement | Plantago | Cut raw material | - |
| P1 | *Rhodiola rosea* root | Poland | China | Dietary supplement | MedFuture | Powder | - |

## Supplementary Figures


**Fig. S1:** HPTLC chromatograms of the *R. rosea* dietary supplements (ethanolic extracts), separated on HPTLC plates silica gel F_254_., using ethyl acetate – methanol – water - formic acid (77:13:10:2, *v/v/v/v*) mobile phase and detected after derivatization at 366 nm. Z1A-Z4A – unprocessed raw material of *R. rosea* roots/rhizomes; K1A-K4A – capsules, P1A – powder, T1A-T3A – tablets, RSV – rosavin.

**Fig S2:** HPTLC chromatograms of the *R. rosea* dietary supplements (infusions), separated on HPTLC plates silica gel F_254_., using ethyl acetate – methanol – water - formic acid (77:13:10:2, v/v/v/v) mobile phase and detected before derivatization at A) 254 nm B) 366 nm C) white light illumination and (D) after derivatization at 366 nm. Z1-Z4 – unprocessed raw material of *R. rosea* roots/rhizomes; K1-K4 – capsules, P1 – powder, T1-T3 – tablets, RSV– rosavin.


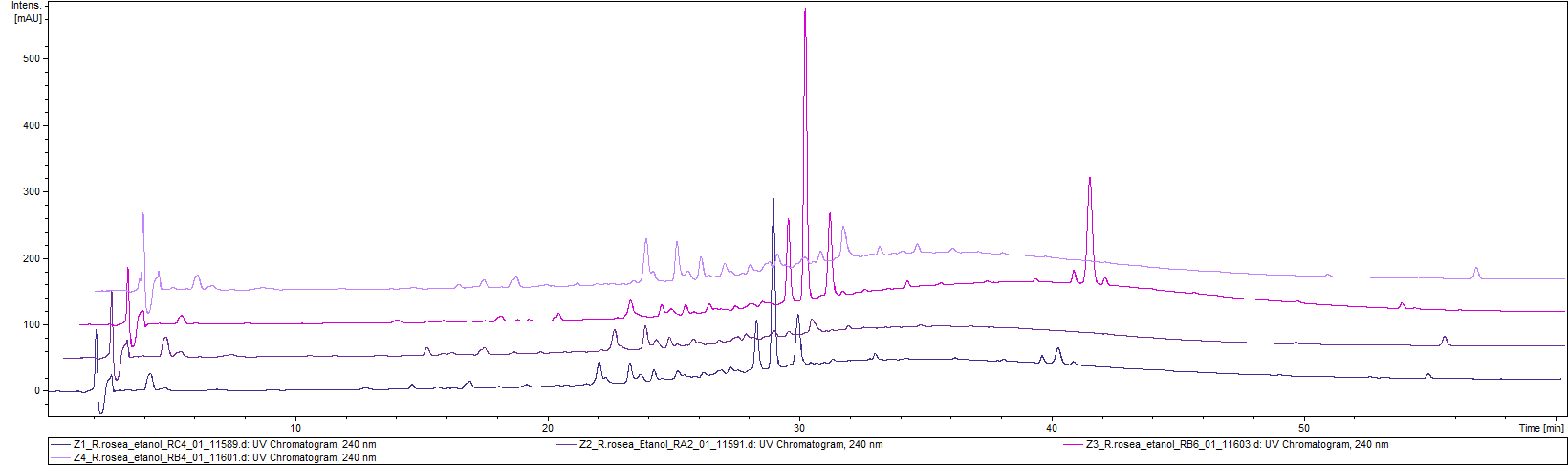


**Rosavin**

**Fig. S3.** LC-DAD-MS/MS chromatograms of 60% ethanolic extracts prepared from the tested cut raw materials*,* recorded at 240 nm. Rosavin was used as a marker compound.

**Cytotoxicity of *R. rosea* root extracts**

**Figure S4:** The influence of tested extracts (50 μg/mL) and rosavin (0.1-5 μM), on the cell viability of BV2 microglia evaluated by MTT assay. Data from three separate experiments assayed in duplicate are expressed as mean ± SEM. TRITON-X 100 0.1% (TX 0.1%) was used as a positive control. Absorbance values for all samples were expressed as percentages relative to the LPS-stimulated control (KST), which was set at 100%. Statistical significance: *** *p* < 0.001 *vs.* stimulated control (KST).

**Figure S5:** The influence of tested extracts (50 μg/mL) and rosavin (0.1-5 μM), on the cell viability of BV2 microglia evaluated by NRU assay. Data from three separate experiments assayed in duplicate are expressed as mean ± SEM. TRITON-X100 0.1% (TX 0.1%) was used as a positive control. Absorbance values for all samples were expressed as percentages relative to the LPS-stimulated control (KST), which was set at 100%. Statistical significance: *** *p* < 0.001 *vs.* stimulated control (KST).

**
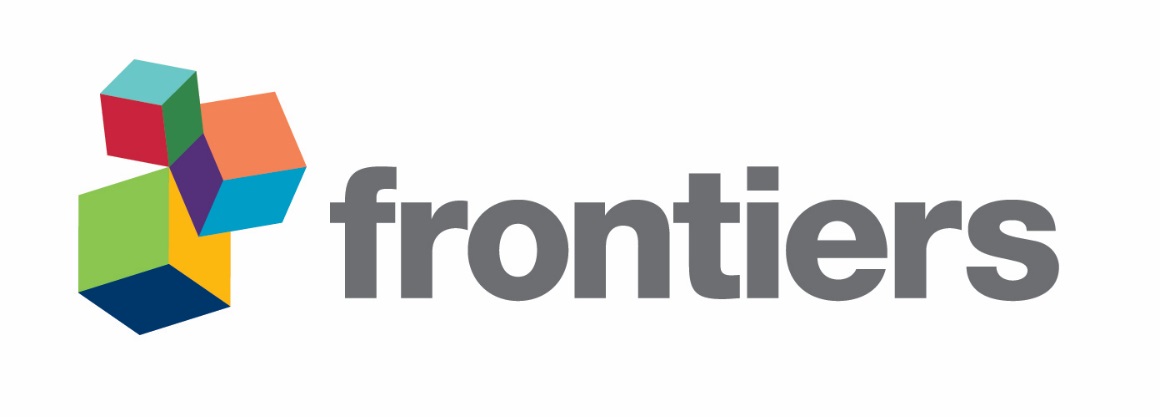
**
